# Rapid implementation of a support intervention for bereavement at the beginning of the COVID-19 pandemic

**DOI:** 10.1192/j.eurpsy.2021.287

**Published:** 2021-08-13

**Authors:** J. Mallet, F. Dousset, M. Colle, H. Cardot, E. Kiesmann, Y. Le Strat, C. Dubertret

**Affiliations:** 1 Psychiatry, LOUIS MOURIER Hospital, Colombes, France; 2 Psychiatry, AP-HP, Louis Mourier Hospital, Colombes, Université de Paris, Faculté de médecine. INSERM UMR1266, Institute of Psychiatry and Neuroscience of Paris, France, COLOMBES, France

**Keywords:** covid-19, bereavement, grief, family support

## Abstract

**Introduction:**

There have been over 900,000 deaths from COVID-19, with more than 3 million people bereaved. These deaths are associated with factors leading to poor bereavement outcomes, and distress in frontline-staff

**Objectives:**

to (i)present the rapid implementation of an intervention for bereavement support; (ii)characterize first calls and follow-up.

**Methods:**

We recruited a multidisciplinary team and prepared a structure called “SIB” (Support and Intervention for Bereavement) in a matter of days. There were three steps for the support (Screening, First-line intervention, Second-line intervention (short follow-up). We collected data screening risk factors for complicated grief (CG).

**Results:**

Between March 24th-May 14th (lockdown, March 16th-May 13th), the hotline received nineteen calls for an intervention. The hospital contacts were various, including mortuary. Fifteen relatives were followed, among them thirteen bereaved for ten deaths (on 52 deaths=19.23%). Dead persons were young (m=59.68 years-old, SD=15.25). All contacts reported several risk factors for a CG (no “goodbye” (100%), no funeral rituals (82.35%)). Six relatives were addressed for short follow-up.

**Conclusions:**

The actual pandemic is at high risk for complicated grief and may until 2021. We hope that all hospitals would implement basic bereavement outreach programs to prepare families for the death and to support them afterwards, as well as provide basic support to frontline staff.

**Disclosure:**

No significant relationships.

